# Monocytic-Myeloid Derived Suppressor Cells of HIV-Infected Individuals With Viral Suppression Exhibit Suppressed Innate Immunity to *Mycobacterium tuberculosis*

**DOI:** 10.3389/fimmu.2021.647019

**Published:** 2021-04-28

**Authors:** Priyanka Namdev, Shiv Patel, Brandi Sparling, Ankita Garg

**Affiliations:** ^1^ Department of Infectious Diseases, College of Veterinary Medicine, University of Georgia, Athens, GA, United States; ^2^ Franklin College of Arts and Sciences, University of Georgia, Athens, GA, United States

**Keywords:** HIV-1, myeloid derived suppressor cell, IL-27, *M tuberculosis*, people living with HIV (PLWH), innate immunity

## Abstract

Tuberculosis can occur during any stage of Human Immunodeficiency virus 1 (HIV) -infection including times when CD4^+^ T cell numbers have reconstituted and viral replication suppressed. We have previously shown that CD11b^+^CD33^+^CD14^+^HLA-DR^-/lo^ monocytic myeloid-derived suppressor cells (MDSC) persist in HIV-infected individuals on combined anti-retroviral therapy (cART) and with virologic suppression. The response of MDSC to *Mycobacterium tuberculosis* (Mtb) is not known. In this study, we compared the anti-mycobacterial activity of MDSC isolated from HIV –infected individuals on cART with virologic suppression (HIV MDSC) and HIV-uninfected healthy controls (HIV (-) MDSC). Compared to HIV (-) MDSC, HIV MDSC produced significantly less quantities of anti-mycobacterial cytokines IL-12p70 and TNFα, and reactive oxygen species when cultured with infectious Mtb or Mtb antigens. Furthermore, HIV MDSC showed changes in the Toll-like receptor and IL-27 signaling, including reduced expression of MyD88 and higher levels of IL-27. Neutralizing IL-27 and overexpression of MyD88 synergistically controlled intracellular replication of Mtb in HIV MDSC. These results demonstrate that MDSC in fully suppressed HIV-infected individuals are permissive to Mtb and exhibit downregulated anti-mycobacterial innate immune activity through mechanisms involving IL-27 and TLR signaling. Our findings suggest MDSC as novel mediators of tuberculosis in HIV-Mtb co-infected individuals with virologic suppression.

## Introduction

Infection with *Mycobacterium tuberculosis* and human immunodeficiency virus-1 (HIV) constitute major burdens of infectious disease in resource-limited countries (1, 2). Co-infection with HIV increases the risk of developing tuberculosis (TB) between 16-27 times (1, 3). It is intriguing that TB can occur in the settings of HIV at any disease state and irrespective of CD4^+^ T-cell numbers (4–8). The profound immune suppression during HIV-infection is considered critical for increased risk of developing TB. Paradoxically, exaggerated proinflammatory responses to *M tuberculosis* in HIV-infection remain inefficient to control TB (9–11). The loss of *M tuberculosis* specific CD4^+^ T-cells and inability of producing perforin by CD8^+^ T-cells during acute HIV infection (12–14), and impaired phagolysosomal fusion, autophagy mediated inhibition of *M tuberculosis* and impaired generation of antimicrobial peptide and mycobactericidal reactive radicals in HIV- *M tuberculosis* co-infected macrophages are considered to increase the risk of TB (15–20). However, explanation for the impaired immunity to *M tuberculosis* in clinically recovered HIV patients remains unresolved.

Increasing evidence establish accumulation of CD11b^+^CD33^+^CD14^+^HLA DR^-/lo^ monocytic myeloid derived suppressor cells (MDSC) in inflammatory clinical conditions (21–24). Besides suppressing T-cell functions, MDSC down regulate antigen presentation, IL-12 production, and augment IL-10 production from macrophages (25–28). Previously we showed that MDSC expand in HIV-infected individuals with replicating virus in an IL-6 dependent manner (29), and continue to remain elevated in HIV (+) individuals on combined antiretroviral therapy (cART), with suppressed viral replication and reconstituted CD4^+^ T-cell numbers (30, 31). Immune activation and hyper-inflammation characterized by elevated levels of soluble tumor necrosis factor receptor II (TNF-RII), neopterin, soluble IL-2R, sCD163, sCD14, IL-6, IP-10 predict disease progression in untreated HIV infection and mediate expansion of MDSC. Of note, IL-6 levels remain elevated in HIV-infected individuals despite successful cART and is responsible for sustained increase in MDSC number as compared to healthy controls. Recent animal and human studies establish peripheral and tissue accumulation of MDSC during infection with Mtb (25, 32, 33). These MDSC are permissive to Mycobacterium and suppress T cell responses to Mtb antigens, thus contributing to TB disease progression. The anti-Mtb innate immunological response of MDSC in HIV-infected persons with virological suppression remains unknown.

In the present study, we compared the anti- *M tuberculosis* activity of MDSC isolated from HIV uninfected (HIV -) and HIV-infected (HIV +) individuals on cART with undetectable HIV RNA copies and recovered CD4^+^ T-cells. We found that compared to MDSC isolated from HIV (–) individuals (HIV (-) MDSC), MDSC isolated from HIV(+) (HIV MDSC) are defective in innate immune mediated control of Mtb. We further show that HIV MDSC have a higher expression of IL-27 receptor (IL-27R); thus IL-27-IL27R axis increase the risk of mycobacterial disease in HIV-infected individuals.

## Materials and Methods

### Patient Groups

Blood was obtained after written informed consent from Quantiferon®-TB negative HIV-uninfected (HIV-) and HIV-infected (HIV+) persons on cART with virologic suppression and CD4^+^. All studies were conducted in accordance with the Declaration of Helsinki guidelines and approved by Institutional Review Board of the University of California San Diego and Institutional Review Board of the University of Georgia Athens.

### Antibodies and Other Reagents

For flow cytometry, Alexa Fluor 488/anti-CD11b, APC or Alexa Fluor 700/anti-CD33, BV605/anti-CD3, BV510/anti-CD19, PerCPCy5.5/anti-CD66b, PE/Cy7/anti-CD14, PE/Dazzle594/anti-HLA DR (all from Biolegend); APC-eFluor 780/anti-CD11b, PE/anti-TLR2, Alexa Fluor 488/anti-TLR4 (all from Thermo Fisher Scientific) and APC/anti-IL27R from R&D systems were used. For live/dead staining we used LIVE/DEAD™ Fixable Aqua Dead cell stain, and for reactive oxygen species CellROX™ Deep Red Reagent, both from Thermo Fisher Scientific. For immunoblotting, anti- IL-27A (Abcam), phosphorylated (p) NF-κB, NF-κB, STAT1 and pSTAT1 (all from R&D Systems), STAT3 (Novus) pSTAT3 and GAPDH (Cell Signaling Technology) were used.

### Isolation and Culture of CD11b^+^CD33^+^CD14^+^HLA-DR^-/lo^ (MDSC) and CD11b^+^CD33^+^CD14^+^HLA-DR^+/hi^ (Monocytes)

Peripheral blood mononuclear cells were stained for CD11b, CD33, CD14, HLA-DR; CD11b^+^CD33^+^CD14^+^HLA DR^-/lo^ MDSC and CD11b^+^CD33^+^CD14^+^HLA DR^+/hi^ monocytes were isolated using Beckman Coulter MoFlo flow cytometer; sorted cells were >90% positive (**Supplemental Figure 1**). Isolated cells (50, 000 – 80, 000) were cultured in RPMI 1640 (Gibco) and 10% human serum (MP Biomedicals) at 37^0^C and 5% CO_2_ in the presence or absence of, *M tuberculosis* Erdman (Erdman) or *M tuberculosis* H37Rv whole cellular lysate (WCL) (10 µg/ml) (BEI resources) for 24 hrs. Supernatants and cells in Trizol were stored at -80°C for cytokine measurement and RNA purification, respectively. For some experiments, cells were cultured in the absence or presence of recombinant IL-27 (rIL-27) (10 ng/ml; R&D Systems).

### Infection of Cells and Measurement of Intracellular Mycobacterial Growth

MDSC (0.1 x 10^5^/well) were plated in 48-well plates in antibiotic free RPMI 1640 medium and 10% human serum. Cells were infected with Erdman at a multiplicity of infection (MOI) of 1:5 for 3 hrs; subsequently, cells were washed and treated with Gentamycin Sulfate (30 µg/ml; VWR Life Sciences) for additional 2-hours to kill extracellular bacteria, and cultured in RPMI 1640 with 10% human serum. Cells were lysed with 0.1% SDS at day-0 and day -3 or day -5 post-infection, and cellular lysates were serially diluted and plated in triplicate on Middlebrook 7H10 agar supplemented with OADC enrichment. The number of colonies were counted after 3-weeks and colony forming units (CFU)/ml determined.

### Overexpression of MyD88

MyD88 was overexpressed in MDSC by transfecting pUNO1-hMYD88 (InvivoGen) expression plasmid using Lipofectamine 3000 (from Thermo Fisher Scientific). Briefly, 1µg MyD88 or empty vector plasmid DNA was diluted in P3000™ reagent and Lipofectamine 3000 in Opti-MEM medium. Diluted plasmid and Lipofectamine were mixed in 1:1 ratio, and incubated for 15 min at room temperature. The plasmid-lipid complex was added to cells and incubated at 37°C and 5% CO_2_.

### Immunolabelling, Flow Cytometry and ROS

Cells were surface stained for CD11b, CD33, CD14, HLA-DR, using cell staining buffer and respective antibodies. Controls for each experiment included unstained cells and fluorescence minus one (FMO) (29).

For ROS, sorted MDSC were infected with Green Fluorescence Protein expressing Mtb Erdman (GFP-Mtb) at MOI of 1:5 for 2-hrs. During the last 30-min of infection, cells were incubated with 1µM of CellROX™ Deep Red reagent at 37°C/5% CO_2_ and subsequently stained with aqua fluorescent LIVE/DEAD fixable dye. All the procedures were performed in Biosafety Level-3 and samples were analyzed on flow cytometer following the institutional biosafety guidelines. Dead cells were excluded and expression of CellROX was analyzed in GFP^+^ Mtb cell gate; Net ROS expression= [Mean fluorescence intensity of ROS by GFP^+^ Mtb MDSC – Mean fluorescence intensity of ROS by uninfected MDSC] (34).

### Immunoblotting

Immunoblotting of cell lysates was performed as previously described (30). Relative densities for target protein bands IL-27A (molecular weight 27 kDa), NF-κB p65/phosphorylated (p^S536^) NF-κB p65 (molecular weight 65 kDa), STAT1/p^Y701^STAT1 (molecular weight 90 kDa) or STAT3/p^Y705^STAT3 (molecular weight 95 kDa) to housekeeping GAPDH (molecular weight 37 kDa) bands were compared using ImageJ (NIH). Fold change was determined as: normalized density in unstimulated cells/normalized density in WCL or rIL-27 stimulated cells.

### Quantification of Cytokines

IL-12p70, TNFα, IL-6, IL-23 and IL-1β was determined in the culture supernatants at 24 hrs-post infection using LUMINEX multiplex system (R&D Systems) and custom designed kit. The fold change in cytokine was determined in *M tuberculosis* treated cells as: cytokine by Erdman infected or WCL treated cells/cytokine by uninfected or unstimulated controls. The quantity of IL-27 in the plasma was measured using Duoset ELISA kit (R&D Systems catalog DY2526).

### Quantitative Real Time PCR (qRT PCR)

Total RNA was isolated from MDSC using TRIzol™ reagent (Thermo Fisher Scientific) according to manufacturer’s protocol; 100-250 ng RNA was used in 20 µl of reverse transcription reaction using SuperScript™ IV VILO™ with ezDNAse Reverse Transcription kit (Thermo Fisher Scientific). TaqMan Gene expression Assay (Thermo Fisher Scientific) were used for qPCR analysis (**Table 1**). The changes in the threshold cycle (C_T_) were calculated by the equation Δ C_T_ = C_T,target_- C_T18S_ for control and WCL stimulated cells, ΔΔC_T_= ΔC_T,WCL_-ΔC_T, control_. The fold change was calculated as $2^{-(△△{\text{C}}_{\text{T}})}$.

**Table 1:** TaqMan Gene Expression Assays (Life Technologies) used for RT PCR.

Gene	TaqMan Assay
**18S RNA**	Hs99999901_s1
**MyD88**	Hs01573837_g1
**NFκB1A**	Hs00355671_g1

### Statistical Analysis

Data are expressed as mean values ± standard error mean (SEM). Paired Student’s t-tests were used to determine the statistical significance for *in vitro* experiments. Comparisons between HIV (-) and HIV (+) using Mann-Whitney *U* test. Statistical analysis was performed using Graphpad Prism 8 (La Jolla, CA). P-values of <0.05 were considered statistically significant.

## Results

### Innate Immune Activity of MDSC in Response to *M tuberculosis*

**Figure 1:**
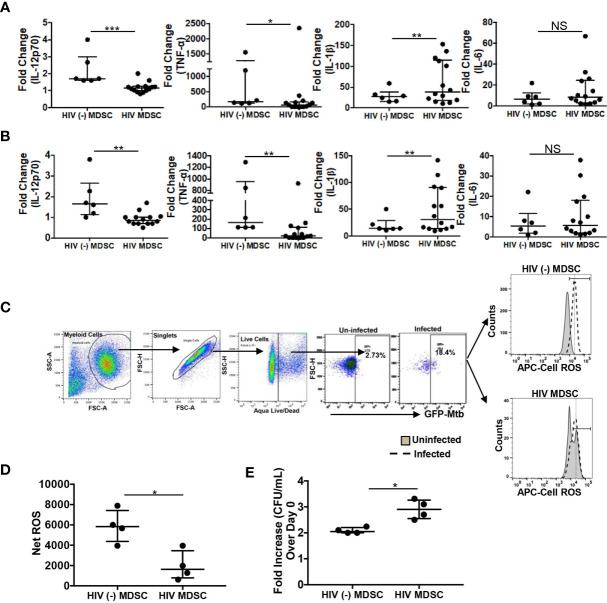
Suppressed innate immunity of HIV MDSC in response to *M tuberculosis*. PBMC from HIV (-) and HIV (+) donors on cART and with virologic suppression was surface stained with anti-CD -11b, -33, -14 and HLA DR antibodies. CD11b^+^CD33^+^CD14^+^HLA DR^-/lo^ MDSC were isolated by flow cytometry and named as HIV (-) MDSC and HIV MDSC, respectively. **(A, B)** Cells were either infected with *M tuberculosis* **(A)** or cultured with *M tuberculosis* whole cell lysate **(B)**. The amount of cytokines were measured in the culture supernatants and fold change was calculated as Quantity in presence of *M tuberculosis*/Quantity in absence of *M tuberculosis*. **(C, D)** MDSC were infected with GFP expressing *M tuberculosis* (Mtb-GFP) at multiplicity of infection (MOI) 1:5 for 2-hrs. During last 30-mins of infection 1μM of CellROX deep read reagents was added and subsequently stained with aqua fluorescent LIVE/DEAD stain. Expression of ROS (CellROS) in Mtb-GFP+ Live cells was determined by flow cytometry (MFI, Mean Fluorescence Intensity). Net ROS expression was calculated as in Materials and Methods. Representative flow cytometry plot of one HIV (-) MDSC and HIV MDSC donor is shown; vertical line is median. **(E)** MDSC cells were infected with *M tuberculosis* at MOI of 1:5 for 3-hrs, washed with PBS to remove extracellular bacteria, *M tuberculosis* growth (colony forming units (CFU)/ml) was determined in the cellular lysates at day-0 and day-5 post-infection. The fold increase in intracellular bacterial replication was measured as CFU/ml at Day-0/CFU/ml at Day-5. For **(A, B, D, E)**, each dot representing an individual donor include observations from 25^th^ to 75^th^ percentile; the horizontal line represents the median value. *p < 0.05, **p < 0.005, ***p < 0.0005. NS, Non Significant.

We have previously established that HIV-infected person with virologic suppression as a result of successful cART exhibit increased circulating numbers of MDSC which regulate immune response to HIV-associated opportunistic pathogen (30). Herein we sought to investigate and compare the response of HIV (-) MDSC and HIV MDSC to *M tuberculosis*. For this, in the initial set of experiments we isolated HIV (-) and HIV MDSC, *in vitro* infected them with *M tuberculosis* or stimulated with *M tuberculosis* whole cell lysate antigen (WCL) and measured cytokines in the culture supernatants; the fold change in response to *M tuberculosis* or WCL was determined. The sorted MDSC were >95% pure (**Supplemental Figure 1**). Similar to a previous report, we found HIV MDSC produced inflammatory cytokines in response to *M tuberculosis*; however, compared to HIV (-) MDSC, fold increase in IL-12p70 and TNFα quantities produced by HIV MDSC infected with live *M tuberculosis* was less (1.2 ± 0.1 vs 2.2 ± 0.4; p=0.001 for IL-12p70, and 248.5 ± 166 vs 556.4 ± 265.4; p=0.04 for TNFα). However, the fold increase in IL-1β produced by HIV MDSC as compared to HIV (-) MDSC was more (61.7±32 vs 29.4±6.5; p=0.02). IL-6 produced by HIV MDSC was also higher than HIV (-) MDSC, but was not significant (15.3 ± 9.6 vs 7.4 ± 4; p=0.6) (**Figure 1A**). Importantly, similar pattern was observed when these cells were cultured with WCL (**Figure 1B**) which further suggests that HIV MDSC are responsive to *M tuberculosis* antigens and do not require active mycobacterial replication for downregulated cytokine production. Of note, quantities of anti-mycobacterial cytokines TNFα and IL-12p70 produced by HIV MDSC was significantly less than that produced by HLA DR^hi^ monocytes (**Supplemental Figures 2A, B**). Collectively, these findings suggest that despite preservation of CD4^+^ T cells and virologic suppression, HIV MDSC exhibit dysregulated anti- *M tuberculosis* cytokine response.

Reactive oxygen species (ROS) is critical for the control of *M tuberculosis* early in infectious process, and ROS production by myeloid cells is regulated by cytokines in TLR-dependent manner (35–39). Therefore, we next compared intracellular ROS produced by *M tuberculosis* infected HIV (-) MDSC and HIV MDSC. For these studies we used GFP-Mtb and measured cellular ROS by flow cytometry. Similar to our previous findings (29), HIV MDSC as compared to HIV (-) MDSC produced more ROS (**Supplemental Figure 2C**). However, the expression of ROS in GFP-Mtb^+^ HIV MDSC was less when compared to HIV (-) MDSC (1957.3 ± 721 vs 5880 ± 807; p=0.04) (**Figures 1C, D**). All together, these findings establish that HIV MDSC exhibit downregulated innate immune response to *M tuberculosis*.

### Increased Intracellular Replication of *M tuberculosis* in MDSC

Our studies demonstrate MDSC isolated from HIV-infected individuals exhibit downregulated anti-mycobacterial innate immune response. Therefore, in the next set of experiments we determined and compared the intracellular growth of *M tuberculosis* in HIV (-) MDSC and HIV MDSC. As compared to day-0, a 3- fold higher CFU of *M tuberculosis* was found in the cellular lysates of HIV MDSC and 2- fold in HIV (-) MDSC at day -5 post-infection (p.i.) (3.0 ± 0.2 vs 2.0 ± 0.06; p=0.02) (**Figure 1E**). These findings suggest that despite virologic suppression, HIV MDSC are inefficient to control *M tuberculosis* replication.

### MDSC Exhibit Truncated TLR Signaling

**Figure 2:**
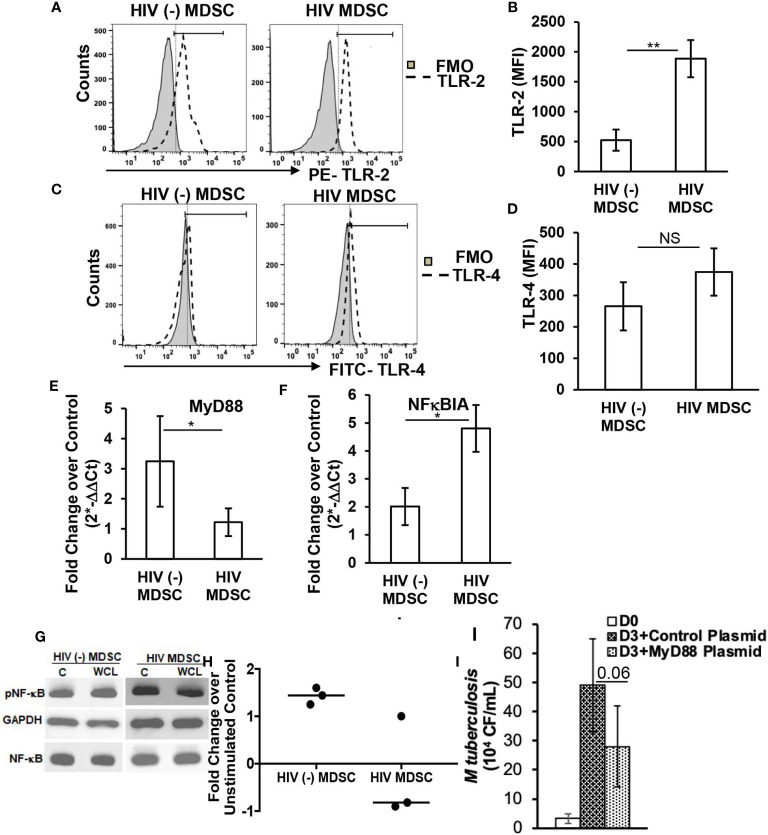
HIV MDSC exhibit truncated TLR signaling. HIV (-) MDSC and HIV MDSC were isolated as in **Figure 1**. MDSC were surface stained with **(A, B)** anti- TLR2 and **(C, D)** anti-TLR4 antibodies. The expression of TLR-2 and -4 was determined by flow cytometry and Mean Fluorescence Intensity (MFI) calculated. Representative flow cytometry plots of TLR-2 and -4 are shown; vertical line is median. **(E, F)** MDSC were cultured without or with *M tuberculosis* WCL for 24-48 hours, expression of housekeeping gene 18S RNA and MyD88 **(E)** and NFκBIA **(F)** and was determined using TaqMan Gene expression assay, and ΔΔC_T_= ΔC_T,WCL_-ΔC_T,control._ The fold change was calculated as $2^{-(△△C_{T})}$. **(G, H)** Total cellular lysates of MDSC cultured without or with *M tuberculosis* WCL were prepared and immunoblotted using anti-GAPDH, -total NFκB p65 and –p^(S536)^NFκB p65 antibody. Fold change was calculated as pNFκB p65 expression in *M tuberculosis* WCL stimulated cells/pNFκB p65 expression in unstimulated cells. Representative Immunoblot of one HIV (-) and HIV (+) donor is shown **(G)**. **(I)** 0.5 x 10^6^ MDSC were transfected with empty vector (Control plasmid) or MyD88 containing expression plasmid (MDSC) using Lipofectamine 3000 reagent. After 16-18 hrs post-transfection, DR^hi^ monocytes and MDSC were infected with *M tuberculosis* at MOI of 1:5 and *M tuberculosis* growth (colony forming units (CFU)/ml) was determined in the cellular lysates at day-0 and -3 post-infection (p.i.). For **(B, D)** histograms are shown for N=9 HIV (-) and N= 17 HIV (+) donors; for **(E, F)** histograms are shown for N= 6 HIV (-) and N= 8 HIV (+) donors; for **(H)** dot represents an individual donor; horizontal line represents the median; for **(I)** histograms shown are for N=3 donors. All histograms show mean values +/- SEM. *p < 0.05, **p < 0.005. NS, Non Significant.

To determine if the reduced anti-mycobactericidal activity and cytokine response of HIV MDSC observed in **Figures 1A–E** is due to the defect in TLR signaling (40–42), we evaluated the surface expression of TLR-2 and -4 on HIV MDSC and compared their expression on HIV (-) MDSC, by flow cytometry. The expression of TLR-2 on HIV MDSC was higher as compared to its expression on HIV (-) MDSC (MFI 1887.3 ± 311.3 vs 525 ± 177.7; p=0.004) (**Figures 2A, B**). However, the expression of TLR-4 on HIV MDSC and HIV (-) MDSC (MFI 374.7 ± 75.4 vs 266 ± 77; p=0.24) (**Figures 2C, D**) was comparable. Unlike TNFα and IL-12p70 cytokine quantities, the expression of TLR-2 and -4 was comparable on HIV MDSC and HIV HLA DR^hi^ monocytes (**Supplemental Figures 3A, B**)

Despite a higher expression of TLR-2, HIV MDSC produced less anti- *M tuberculosis* cytokines, we questioned if the differences occur in the expression of cytoplasmic adapter protein MyD88 (42, 43). Since our cytokine studies (**Figures 1A, B**) demonstrate similar anti-mycobacterial activity in response to infectious or non-infectious *M tuberculosis*. Therefore, for gene expression assays, we cultured these cell types with WCL for 24-hrs and quantified messenger RNA (mRNA) for MyD88 by qRT PCR. HIV (-) MDSC cultured with WCL exhibited a 3.3-fold increased expression of MyD88 as compared to unstimulated controls; the expression of MyD88 in HIV MDSC cultured with WCL was less than HIV (-) MDSC (1.22 ± 0.5 vs 3.3 ± 1.5; p= 0.04) (**Figure 2E**) and HIV HLA DR^hi^ monocytes (**Supplemental Figure 3C**). Downstream of TLR-signaling and pivotal for the synthesis of proinflammatory cytokines and ROS, is the activation and nuclear transport of the transcription factor NF-κB, which is regulated by the degradation of NFκB-inhibitor-α (NFκBIα) encoded protein IκBα (42, 43). An increase in NFκBIα expression inhibits the degradation of IκBα thus sequestering NF-κB in the cytoplasm and inhibition in proinflammatory cytokine production (37–39). In order to establish that reduced quantities of cytokine produced by HIV MDSC is due to insufficient NF-κB activation, we quantified mRNA for NFκBIα in response to WCL by RT PCR in HIV MDSC and HIV (-) MDSC. HIV MDSC compared to HIV HLA DR^hi^ monocytes (**Supplemental Figure 3D**), and HIV (-) MDSC exhibited a 5-fold increased expression of NFκBIα in response WCL (4.8 ± 0.8 vs 2.1 ± 0.7; p= 0.05) (**Figure 2F**). This was accompanied by less phosphorylation of NF-κB p65 in HIV MDSC cultured with WCL (**Figures 2G, H**).

Next, we overexpressed MyD88 by transfecting expression plasmid containing open reading frame of human MyD88 in HIV MDSC and measured the intracellular replication of *M tuberculosis* at 72-hrs post-infection. Compared to MDSC transfected with control plasmid, MyD88 transfection decreased the intracellular replication of *M tuberculosis*, but this was not significant (50 x 10^4^ ± 17 x 10^3^ vs 28 x 10^4^ ± 14 x 10^3^ CFU/ml, p=0.06) (**Figure 2I**). Collectively, these studies establish that the impaired anti-mycobacterial innate immune response of HIV MDSC is due to the reduced expression of MyD88, and reconstituting it partially restores the anti-mycobacterial function of HIV MDSC.

### IL-27 and MDSC

**Figure 3:**
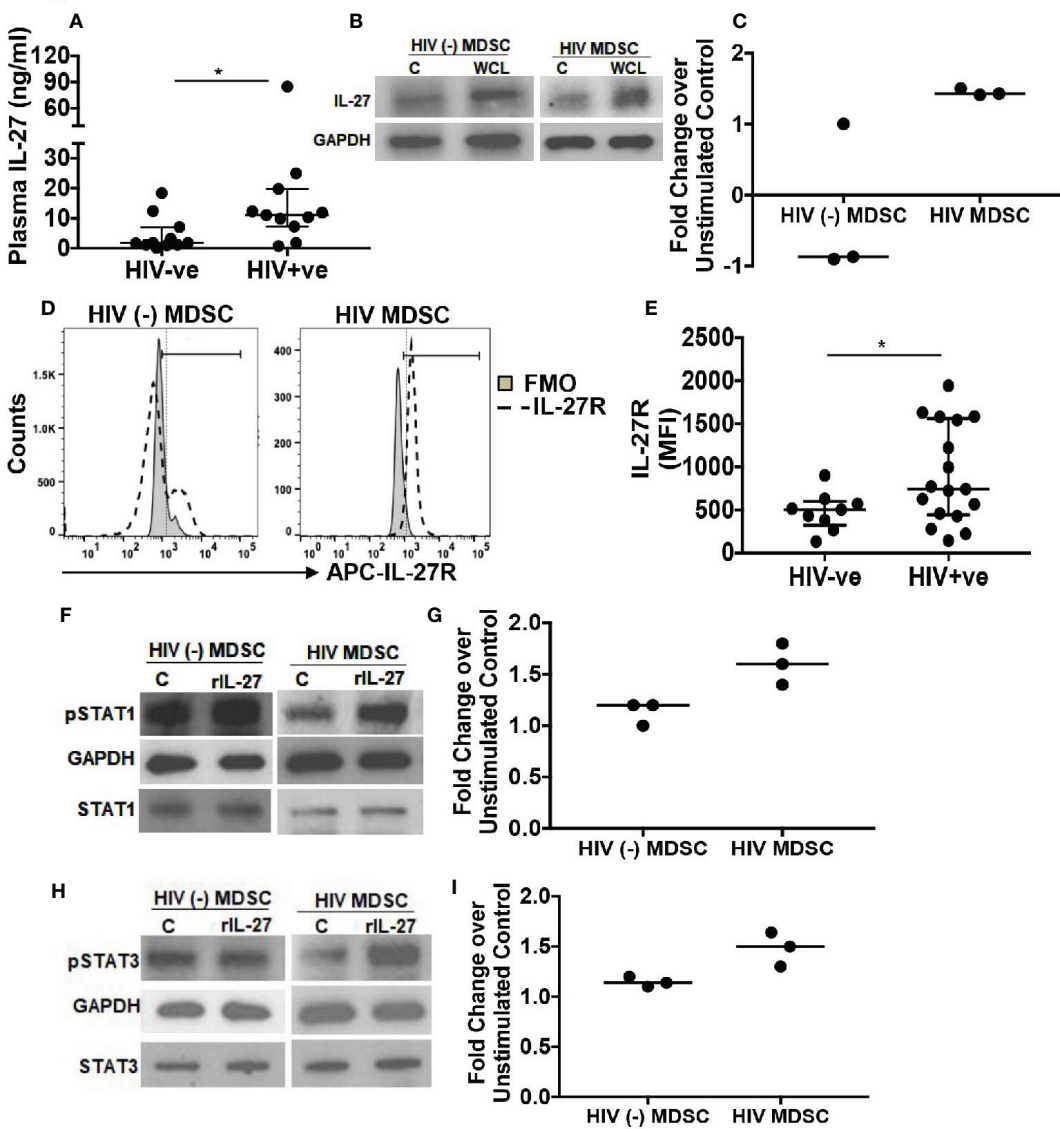
Increased expression of IL-27 and IL-27R by HIV MDSC. To determine the role of IL-27, **(A)** IL-27 was quantitated in the plasma of HIV (-) and HIV (+) individuals with virologic suppression by ELISA. **(B, C)** HIV (-) MDSC and HIV MDSC were isolated and cultured without or with *M tuberculosis* WCL. Total cellular lysates were immunoblotted using anti-GAPDH and –IL-27A antibodies to determine the expression of IL-27p28. Representative Immunoblot of one HIV (-) MDSC and HIV MDSC donor is shown; vertical line is median **(B)**. **(D, E)** MDSC were isolated and surface stained with anti-IL-27 receptor (IL-27R) antibody. The expression of IL-27R was determined by flow cytometry and Mean Fluorescence Intensity (MFI) calculated. Representative flow cytometry plot is shown **(D)**. **(F–I)** To determine functional response of IL-27R, MDSC were isolated and stimulated with recombinant IL-27 (rIL-27) for 30-minutes. Total cellular lysates of unstimulated and rIL-27 stimulated cells were immunoblotted using anti-GAPDH, -total STAT1, -total STAT3, -p^(Y701)^STAT1 and –p^(Y705)^STAT3 antibody. Fold change was calculated as p -STAT1 or –STAT3 expression in rIL-27 stimulated cells/as p -STAT1 or –STAT3 expression in unstimulated cells. Representative Immunoblot of one HIV (-) MDSC and HIV MDSC donor is shown. For **(A, E)**, each dot representing an individual donor include observations from 25^th^ to 75^th^ percentile; the horizontal line represents the median value; for **(C, G, I)** each dot represents an individual donor; horizontal line represents the median. *p < 0.05.

IL-27 is an immune regulatory cytokine that inhibits phagosomal activity of macrophages in response to bacterial infection including Mycobacterium (44, 45). In HIV-infection, we previously showed that the plasma IL-27 level correlates positively with CD4^+^ T-cell count and negatively with HIV- viral load (30). We sought to determine if IL-27 inhibits anti-mycobacterial activity of HIV MDSC. To this end, initially we quantified the amount of IL-27 in the plasma of HIV-infected individuals and in healthy controls. Compared to healthy controls, the quantity of IL-27 in the plasma of HIV-infected individuals was high (4.6 ± 1.7 vs 17.7 ± 7 ng/ml; p= 0.03) (**Figure 3A**). Next, we measured IL-27 in the culture supernatants of HIV MDSC and HIV (-) MDSC infected with *M tuberculosis* or stimulated with WCL as in **Figures 1A, B**; IL-27 in these culture supernatants was undetectable. However, we observed increased expression of IL-27 in the cellular lysates of HIV MDSC *in vitro* stimulated with WCL (**Figures 3B, C**).

Additionally, we measured the surface expression of IL-27R on HIV MDSC and HIV (-) MDSC isolated from HIV-infected and HIV-uninfected individuals, respectively, by flow cytometry. The expression of IL-27R on HIV MDSC was high (MFI 938.5 ± 130 vs 481 ± 73; p= 0.03) (**Figures 3D, E**) as compared to HIV (-) MDSC, and HIV HLA DR^hi^ monocytes (**Supplemental Figure 3E**). IL-27R is indispensable for IL-27 signaling, which induces phosphorylation of STAT1 and STAT3 in myeloid cells (46, 47). In order to establish the functional significance of increased expression of IL27R on HIV MDSC, we stimulated HIV (-) MDSC and HIV MDSC with rIL-27 for 30 mins and assessed pSTAT1 and pSTAT3 in cellular lysates. Compared to unstimulated and rIL-27 stimulated HIV (-) MDSC, HIV MDSC exhibited increased pSTAT1 and pSTAT3 (**Figures 3F–I**) expression. Collectively, these findings suggest that IL-27 may modulate the anti-mycobacterial activity of MDSC in clinically recovered HIV-infected individuals.

### IL-27 Inhibits Anti-Mycobacterial Activity of MDSC

**Figure 4:**
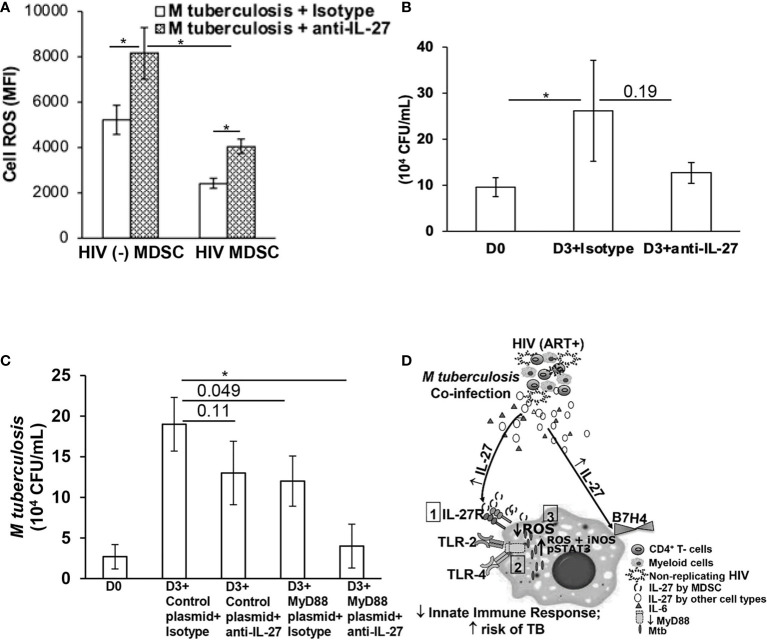
IL-27 inhibits anti-mycobacterial activity of MDSC. To determine that IL-27 inhibits anti-mycobacterial activity of MDSC, HIV (-) MDSC and HIV MDSC were isolated as in **Figure 1** using flow cytometry and **(A)** infected with Mtb-GFP at MOI of 1:5, in the presence of neutralizing IL-27 or isotype matched control antibody for 2-hrs. During last 30-mins of infection 1 µM of CellROX deep read reagent was added and subsequently stained with aqua fluorescent LIVE/DEAD stain. Expression of ROS (Cell ROS) in Mtb-GFP+ Live cells was determined by flow cytometry and Mean Fluorescence Intensity (MFI) calculated. Net ROS expression was calculated as in *Materials and Methods*. **(B)** MDSC were infected for 3-hrs, washed with PBS to remove extracellular bacteria and cultured with neutralizing IL-27 or isotype matched control antibody. *M tuberculosis* growth (colony forming units (CFU)/ml) was determined in the cellular lysates at day-0 and -3 post-infection (p.i.). **(C)** MDSC were transfected with empty (Control) or MyD88 expressing plasmid using Lipofectamine 3000. After 16-18 hrs post-transfection, were infected with *M tuberculosis* at MOI of 1:5 for 3-hrs washed with PBS to remove extracellular bacteria and cultured with neutralizing IL-27 or isotype matched control antibody. *M tuberculosis* growth (colony forming units (CFU)/ml) was determined in the cellular lysates at day-0 and -3 post-infection (p.i.). **(D)** Regulation of immunity in HIV-Mtb co-infection: HIV-infected individuals on cART with suppressed HIV replication have increased IL-27 and MDSC in these individuals exhibit (1) higher expression of IL-27R, (2) lower expression of MyD88 which abrogates TLR-signaling and (3) reduced expression of Reactive Oxide Species (ROS) in response to *M tuberculosis*. The histograms show mean values +/- SEM; N=4 donors. *p < 0.05.

Our initial findings established reduced proinflammatory cytokines, ROS and increased IL-27 by HIV MDSC in response to *M tuberculosis*; we argued if IL-27 suppresses ROS activity. To evaluate this, we infected HIV (-) MDSC and HIV MDSC with GFP-Mtb in the presence or absence of neutralizing IL-27 or isotype matched control antibody and measured intracellular ROS by flow cytometry as in **Figure 1D**. Consistent with our findings of **Figure 1D**, GFP-Mtb+ HIV (-) MDSC compared to HIV MDSC express more ROS (MFI 5209.7 ± 647.4 vs 2407 ± 217; p= 0.05). The expression of ROS increased in both HIV (-) MDSC and HIV MDSC upon neutralization of IL-27 (MFI 5209.7 ± 647.4 vs 8147.7 ± 1138.6; p= 0.02 and 2407 ± 217 vs 4030.7 ± 320; p=0.03, respectively) (**Figure 4A**). Of note, IL-27 neutralization increased the ROS expression of HIV MDSC in response to *M tuberculosis* but its level remained lower than HIV (-) MDSC (MFI 4030.7 ± 320 vs 5209.7 ± 647.4; p= 0.05).

Since overexpression of MyD88 in HIV MDSC partially inhibited intracellular replication of *M tuberculosis* (**Figure 2F**), we further investigated if IL-27 plays a role in anti-mycobacterial activity of MDSC. For this, MDSC isolated from HIV-infected individuals were infected with *M tuberculosis* and treated with neutralizing IL-27 or isotype matched control antibody; intracellular *M tuberculosis* replication was determined at day-3 post-infection. Compared to isotype matched control, lower CFU/ml were found in the cellular lysates of MDSC infected with *M tuberculosis* and treated with neutralizing IL-27 antibody (24 x 10^4^ ± 9 x 10^3^ vs 12 x 10^4^ ± 2 x 10^3^ CFU/ml, p=0.16) (**Figure 4B**), but this remained non-significant.

Next to determine if neutralizing IL-27 and overexpression of MyD88 synergistically affect intracellular replication of *M tuberculosis*, HIV MDSC were transfected with MyD88 expression plasmid, infected with *M tuberculosis* and treated with neutralizing IL-27 antibody. Compared to transfection with control vector and isotype matched antibody treatment, MyD88 overexpression and neutralizing IL-27 inhibited intracellular replication of *M tuberculosis* (16 x 10^4^ ± 4 x 10^3^ vs 2 x 10^4^ ± 0.4 x 10^3^ CFU/ml, p=0.04) (**Figure 4C**). All together, these results establish that IL-27 inhibits anti-mycobactericidal activity of HIV MDSC and contributes to the increased intracellular replication of *M tuberculosis* in MDSC of HIV-infected individuals. These studies further suggest that regulating MyD88 and IL-27 augments anti-mycobacterial activity of HIV MDSC.

## Discussion

In HIV- *M tuberculosis* co-infection, TB can occur at times when CD4^+^ T-cell numbers have reconstituted and viral replication is suppressed (4–6). The increased risk of TB in HIV- *M tuberculosis* co-infection has primarily focused on the defects in CD4^+^ and CD8^+^ T-cell number and function (13, 48, 49). However, myeloid cells (CD14^+^ monocytes/macrophages and alveolar macrophages) are the first cells to interact with *M tuberculosis* and shape T-cell responses; it is important to understand *M tuberculosis* interaction with these cells and the defects in innate immunity (10, 16, 18, 50). Despite virologic suppression and CD4^+^ T-cell recovery, a subset of myeloid cells known as CD14^+^ MDSC are present in HIV-infected individuals that down-regulate T-cell response to cytomegalovirus (30, 31). In this research, we demonstrate that MDSC isolated from HIV (+) individuals with virologic suppression as compared MDSC isolated from HIV (-) controls exhibit suppressed cytokine production in response to *M tuberculosis* or WCL. We also demonstrate that *M tuberculosis* infected HIV MDSC produce less ROS and exhibit a higher intracellular replication. Additionally, we show that the impaired anti-mycobacterial activity of HIV MDSC is through mechanisms involving cytokine IL-27 and truncated TLR-signaling. These support the previous findings demonstrating suppressed innate immune function and increased bacillary load in HIV- *M tuberculosis* co-infected individuals (10, 17, 18, 20).

MDSC expand during various pathological conditions as a result of acute or chronic inflammation and are important mediators of immune suppression (21, 23–26, 29, 31, 32, 51). In murine TB, MDSC are present at the disease site, harbor mycobacteria, produce cytokines TNF-α, IL-1 α and IL-6, and suppress anti-mycobacterial T-cell IFN-γ responses (25, 33). Accumulation of MDSC in lungs heightens TB lethality, and MDSC depletion by all-trans-retinoic acid ameliorates primary progressive TB (25, 52, 53). MDSC also expand in peripheral blood and pleural fluid of TB patients and HIV-*M tuberculosis* co-infected children, and suppress T-cell effector functions through mechanisms involving suppressed TNF-α, IL-2 and IL-10 (25, 54–56). These findings collectively establish downregulated innate immune responses mediated by MDSC contribute to the failure to control *M tuberculosis*. However, we provide the first evidence that MDSC present in HIV-infected individuals with virologic suppression are defective in innate immune mediated control of *M tuberculosis* and are potential mediators of tuberculosis in these individuals. Similar to the previous findings, we also observed that MDSC isolated from HIV-infected individuals phagocytose *M tuberculosis*, thus provide a niche for pathogen survival, increasing the risk of TB (32, 33). Unlike the findings of Agarwal et al, we found higher intracellular replication of *M tuberculosis* and lower quantities of cytokines from HIV MDSC as compared to HIV (-) MDSC (32). Agarwal et al, generated MDSC and monocyte derived macrophages (MDM) *in vitro* to compare the effect of these cell types on *in vitro* granuloma; we have utilized a more physiological *ex vivo* approach to compare the mycobacterial response of MDSC isolated from HIV-infected and -uninfected individuals and did not use any cytokine treatment prior to infection with *M tuberculosis*. These observed differences in the anti-mycobacterial activity of HIV (-) MDSC and HIV MDSC are suggestive of the impact of HIV on cellular reprogramming of MDSC. Our findings are similar to the *in vivo* study of Knaul et al. establishing higher mycobacterial load in MDSC isolated from lungs of *M tuberculosis* susceptible animals (33). A limitation of our study is that we isolated peripheral MDSC from HIV-infected individuals to determine their anti-mycobacterial activity. Nonetheless, presence of arginase-I and nitric oxide co-expressing MDSC like cells in the necrotic granulomas of *M tuberculosis* infected macaques (53, 57), and accumulation of MDSC in blood at advanced stage of *M tuberculosis* infection establish that MDSC are critical mediators of tuberculosis disease pathogenesis and suppress both innate and adaptive immune mediated control of *M tuberculosis* (25, 33, 53). The increased bacillary load observed in HIV MDSC conceivably is due to higher infectivity of these cells. Owing to a small sample size we were unable to statistically determine this aspect. We are currently investigating the interaction and trafficking of *M tuberculosis* inside MDSC of HIV- uninfected and –infected individuals. Here we propose that MDSC in virologically suppressed HIV-infected individuals are permissive to *M tuberculosis* and contribute to increased bacillary load observed in these individuals.

TNF-α and IL12p70 are indispensable to control *M tuberculosis*; these cytokines enhance phagosome-lysosome maturation, antigen presentation and mobilization of activated T cells (58–60). Asymptomatic HIV-infection is associated with reduced release of TNF-α by alveolar macrophages and peripheral blood mononuclear cells in response to *M tuberculosis* infection or immunogenic *M tuberculosis* specific proteins (61, 62). This is a result of impaired nuclear translocation of NF-κB, and HIV Nef mediated destabilization of TNF-α mRNA (61, 62). Accordingly, we found that *M tuberculosis* upregulates NFκBIα in HIV MDSC which masks the nuclear localization signals of NF-κB by stabilizing IκBα. Additionally, both TNF-α and IL12p70 play an important link between innate and adaptive immune responses. Although yet to be investigated, it is plausible that the reduced *M tuberculosis* specific cytokines produced by HIV MDSC results in the decreased expression of chemokines CCL5, CXCL9 and CXCL10 thus restricting the migration of CXCR3^+^, CCR1^+^ and CCR5^+^ activated T cells to granuloma or secondary lymphoid tissues, resulting in loss of immunity in HIV- *M tuberculosis* co-infected individuals (63, 64). We previously reported increased mRNA expression of p47phox subunit of ROS by HIV MDSC, which mediates down regulation of T cell function (29). Consistently, HIV MDSC produced profound ROS at basal level, but not in response to *M tuberculosis* infection. Here we show that the reduced release of anti-mycobacterial cytokines by HIV MDSC directly affects the mycobacterial load through mechanism involving ROS suppression. ROS generated by NADPH oxidase is a vital component of *M tuberculosis* containing mature phagosome, facilitating bacterial killing; ROS also regulates NF-κB and MAP kinase signaling in TLR-dependent manner (36). Our study affirms that truncated TLR-MyD88 axis in HIV MDSC increases mycobacterial load, which potentially amplifies the risk of tuberculosis in HIV patients with virologic suppression; reconstituting MyD88 partially recovers mycobactericidal activity of HIV MDSC. It is possible that TNF-α and IL12p70 produced by macrophages compensates the suppressed anti-mycobacterial activity of HIV MDSC, but inhibition of monocyte/macrophage function by MDSC observed in tumors cannot be ruled out in HIV-*M tuberculosis* co-infection (28, 65).

IL-6 is a pleiotropic proinflammatory cytokine produced by multiple cell types in response to inflammatory stimuli including IL-1β, TNF-α, TLRs, prostaglandins and stress responses (66, 67). IL-6 deficiency leads to impaired innate and adaptive immunity to viral, bacterial and parasitic infection. Even though, the importance of IL-6 during *M tuberculosis* infection is not well understood, its neutralization increases susceptibility to infection and mycobacterial load, while delaying T-cell accumulation and IFNγ expression, both during primary infection, and vaccination with BCG and a subunit vaccine (68–70). Additionally, IL-6 is critical for differentiation and maintenance of IL-23 dependent T_H_17 cells- important for recruitment of neutrophils to infection site, and IFNγ mediated control of *M tuberculosis* (71–73). IL-23 treatment of *M tuberculosis* infected animals reduces mycobacterial burden and augments cellular responses; its absence increases mycobacterial burden, and decreases the expression of IL-17, IL-22 and CXCL13 resulting in accumulation of lymphocytes around the vessels rather than within granulomas (74, 75). While we did not evaluate T-cell responses in this study, we found comparable level of IL-6, but lower level of IL-23 produced by HIV MDSC as compared to HIV (-) MDSC in response to *M tuberculosis* (**Figure 1A** and **Supplemental Figures 4A, B**). Further studies are needed to determine the exact role of HIV MDSC in suppressing adaptive immunity to *M tuberculosis*. Chronic immune activation driven by IL-6 and TNF-α are major predictors of HIV disease progression. Elevated level of IL-6 present in serum, mucosal and lymphoid tissues augment HIV replication through C/EBPb mediated binding to the HIV long terminal repeats and inhibition of APOBEC3G (76–81). We and others have established that IL-6 mediates MDSC expansion, and even though its level decline following successful administration of cART, but still remain elevated as compared to healthy controls thus maintaining increased numbers of MDSC in HIV patients with virologic suppression (29–31). These MDSC increase the risk of tuberculosis in HIV- *M tuberculosis* co-infection.

IL-27 and IL-27R are expressed by activating myeloid cells including dendritic cells and macrophages, and modulate both macrophage and T-cell activity during *M tuberculosis* infection (45, 82, 83). We and others have observed that IL-27 directly inhibits HIV replication (**Supplemental Figures 4C, D**) in PBMC. Although the mechanism of IL-27 mediated viral suppression is not fully established, it appears that the inhibition of spectrin β nonreythrocyte 1 (SPTBN1) by IL-27 plays a critical role (84). In HIV-infected individuals serum IL-27 levels correlate negatively with viral load and positively with CD4^+^ T-cell counts (30), and IL-27 induced IL-6 and TNF-α production is downregulated in HIV infection (85). This is of significance in the settings of HIV-*M tuberculosis* co-infection where IL-27 supports clinical recovery from HIV but is detrimental to control *M tuberculosis*. Previously, Egidio et al. also found increased expression of IL-27 in TB patients co-infected with HIV as compared to latent TB infection in south and southeast African cohorts (82). In this study, we provide the first evidence of the direct effect of IL-27 on *M tuberculosis* in HIV- *M tuberculosis* co-infection. Our findings that neutralizing IL-27 augments the expression of ROS in response to *M tuberculosis* and controls intracellular replication of *M tuberculosis* corroborate with the findings that, IL-27 negatively regulates macrophage response by inhibiting the expression of phagosomal vacuolar ATPase (V-ATPase) and lysosomal integrated membrane protein-1 (CD63), resulting in suppression of phagosomal acidification and cathepsin D maturation, all these lead to increased bacillary load (44, 45). With regard to the expression of IL-27, unlike Egidio et al. and Cory et al, IL-27 transcripts were undetectable in our study (45, 82). The potential reasons for this could be: 1) difference in the samples used, Egidio et al. measured IL-27 gene expression in whole blood RNA (82) and Cory et al. utilized macrophages (45), and 2) the amount of RNA used by Cory et al. for reverse transcription and subsequent transcript analysis was 750 ng; given the low numbers of MDSC that could be isolated from HIV-infected individuals, we were unable to obtain such a high quantity of RNA and thus low copies of IL-27 could be present below our detection limit. Of note, p28 subunit specific to IL-27 is produced by activated myeloid cells through TLR-2, -4 and -9 in MyD88 dependent manner (86), and in MyD88 independent manner through TLR-4 - TIR-domain-containing adapter inducing IFNβ (TRIF) and IFN regulatory factor 3 (IRF3) pathways (87, 88). In this research, we show that HIV MDSC are deficient in MyD88 expression, suggesting that a MyD88-independent mechanism is the major pathway involved in IL-27 synthesis in response to *M tuberculosis*. We are currently investigating the mechanism of IL-27 mediated suppression of innate immunity in response to *M tuberculosis* with or without co-infection with HIV. The increased plasma IL-27 in HIV-infected individuals in this and other studies suggest multiple cell types produce this cytokine and exhibits anti-HIV activity (30, 84). In the present study, we provide the first evidence of increased IL-27 and IL-27R by MDSC in HIV-infected individuals with virologic suppression. We previously showed that IL-27 upregulates B7-H4 expression on MDSC, which regulates T-cell function (30). In this research, we propose that IL-27 in HIV- *M tuberculosis* co-infection acts on MDSC both in autocrine and paracrine manner, and that IL-27-IL27R axis is a potential mediator of suppressed immunological response to *M tuberculosis* (**Figure 4D**).

In summary, our *ex vivo* and *in vitro* data collectively establish that HIV-infected individuals with virologic suppression have increased levels of circulating IL-27 and MDSC. MDSC exhibit truncated TLR-mediated innate immunological function in response to *M tuberculosis*. Further, these MDSC express increased surface expression of IL-27R which may further downregulate the innate immunity to *M tuberculosis*. These findings provide a mechanistic model of how MDSC can increase the risk of tuberculosis in HIV-*M tuberculosis* co-infected individuals. Moreover, our findings suggest that IL-27/IL-27R and MDSC provide attractive biomarkers to assess tuberculosis prognosis during HIV-infection.

## Data Availability Statement

The original contributions presented in the study are included in the article/**Supplementary Material**. Further inquiries can be directed to the corresponding author.

## Ethics Statement

The studies involving human participants were reviewed and approved by The University of California San Diego and The University of Georgia Athens. The patients/participants provided their written informed consent to donate blood for this study.

## Author Contributions

AG developed the study, performed experiments, analyzed data, and wrote the paper. PN, SP and BS performed the experiments, and analyzed the data. All authors contributed to the article and approved the submitted version.

## Funding

This research was supported by AI127132 from the National Institute of Allergy and Infectious Diseases (NIAID) and The University of Georgia Research Foundation.

## Disclaimer

The content is solely the responsibility of the authors and does not necessarily represent the official views of the National Institutes of Health.

## Conflict of Interest

The authors declare that the research was conducted in the absence of any commercial or financial relationships that could be construed as a potential conflict of interest.
