# Supplementary material for: Monocytic-Myeloid Derived Suppressor Cells of HIV-Infected Individuals With Viral Suppression Exhibit Suppressed Innate Immunity to Mycobacterium tuberculosis
Source: Front Immunol. 2021 Apr 28;12:647019. doi: 10.3389/fimmu.2021.647019 (PMC8113814; doi:10.3389/fimmu.2021.647019)
Supplement: Supplementary file 1 [file DataSheet_1.doc]

**Monocytic-Myeloid Derived Suppressor Cells of HIV-Infected Individuals with Viral Suppression Exhibit Suppressed Innate Immunity to *Mycobacterium tuberculosis***

Priyanka Namdev ^1^, Shiv Patel ^2^, Brandi Sparling ^1#^, Ankita Garg^*^

^1^ Departments of Infectious Diseases, College of Veterinary Medicine, University of Georgia, Athens, GA, USA

^2^ Franklin College of Arts and Sciences, University of Georgia, Athens, GA, USA

#Present Address: Western University of Health Sciences, Pomona, USA

*Address correspondence to: Ankita Garg, PhD,

Department of Infectious Diseases

College of Veterinary Medicine

University of Georgia,

Athens, GA, 30606

USA

**Phone**: 706-542-4541

**Fax**: 706-542-5771

**E-mail**: [agarg@uga.edu](mailto:agarg@uga.edu)


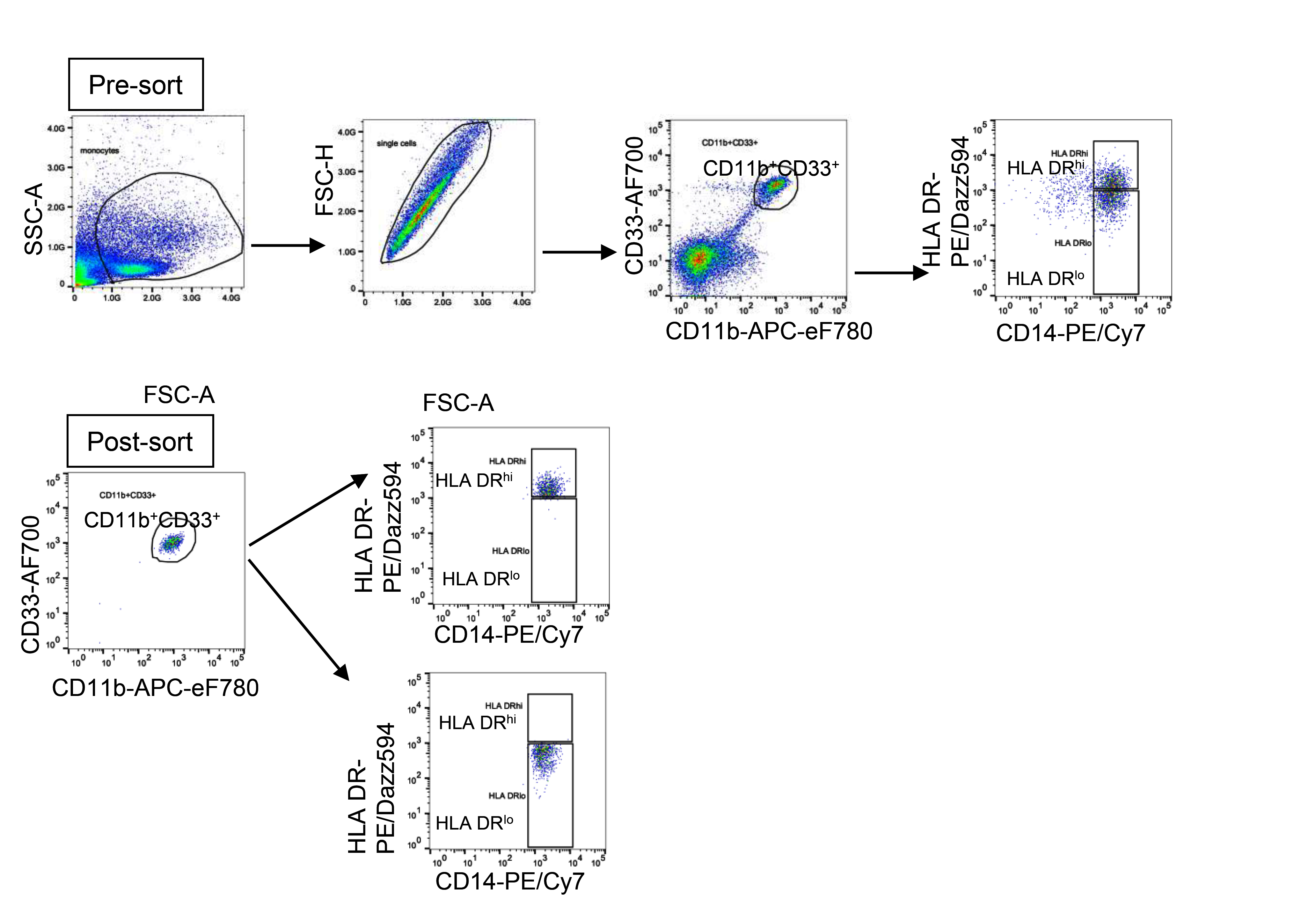
Supplementary Figure 1

Purity of sorted CD11b^+^CD33^+^CD14^+^HLA DR^+/hi^ (HLA DR^hi^) and CD11b^+^CD33^+^CD14^+^HLA DR^-/lo^ (MDSC) isolated from peripheral blood mononuclear cells of HIV-infected individuals on combined anti-retroviral therapy and virologic suppression (HIV MDSC) or healthy controls (HIV (-) MDSC).


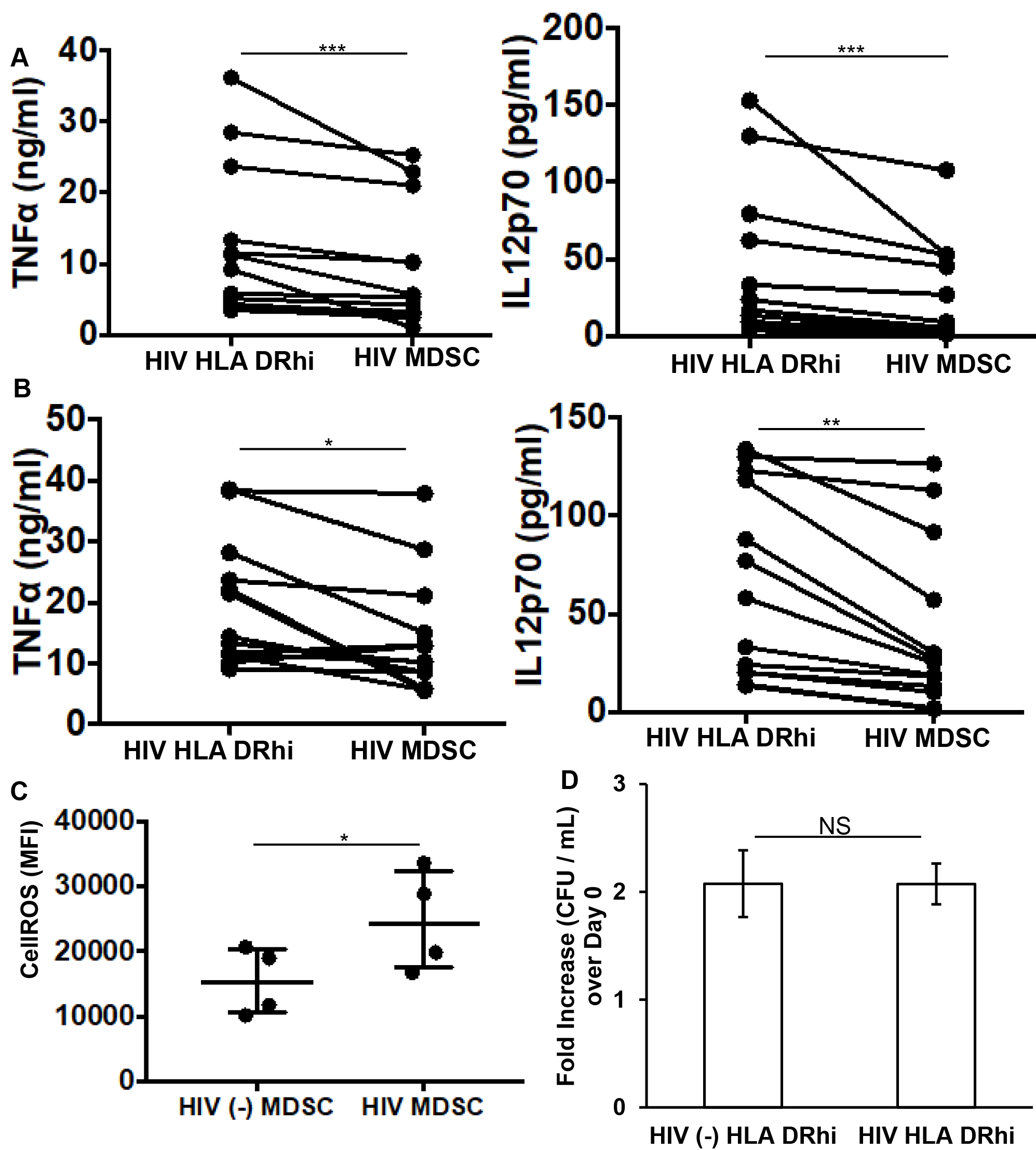
Supplementary Figure 2

**(A-B) HIV MDSC as compared to HLA DR^hi^ cells produce low levels of TNFα and IL-12p70 in response to *M tuberculosis*:** CD11b^+^CD33^+^CD14^+^HLA DR^+/hi^ (HIV HLA DR^hi^) and CD11b^+^CD33^+^CD14^+^HLA DR^-/lo^ (HIV MDSC) were (A) infected with *M tuberculosis* Erdman or (B) cultured with *M tuberculosis* whole cell lysate for 24 hours. The quantities of IL-12p70 and TNFα was measured in the culture supernatant**. (C) Increased expression of ROS in HIV MDSC:** MDSC from healthy (HIV (-) MDSC) and HIV-infected individuals (HIV MDSC) were first stained with CellROX deep read reagent and subsequently with aqua fluorescent LIVE/DEAD stain. The expression of basal level of ROS (CellROS) was determined by flow cytometry (MFI: Mean Fluorescence Intensity). **(D) Comparable replication of intracellular M tuberculosis in HIV (-) and HIV HLA DR^hi^ cells:** CD11b^+^CD33^+^CD14^+^HLA DR^+/hi^ cells isolated from healthy (HIV (-) HLA DR^hi^) and HIV-infected (HIV HLA DR^hi^) individuals were infected with *M tuberculosis* at MOI of 1:5 for 3-hrs, washed with PBS to remove extracellular bacteria, *M tuberculosis* growth (colony forming units (CFU) / ml) was determined in the cellular lysates at day-0 and day-5 post-infection. The fold increase in intracellular bacterial replication was measured as CFU/ml at Day-0 / CFU/ml at Day-5. For (A), (B) and (C), each dot represents an individual donor; for (C) each dot representing an individual donor include observations from 25^th^ to 75^th^ percentile; the horizontal line represents the median value; for (D) histograms shown are for N=4 donors in each group. All histograms show mean values +/- SEM. *p<0.05, **p<0.005, ***p<0.0005, NS: Non-Significant.


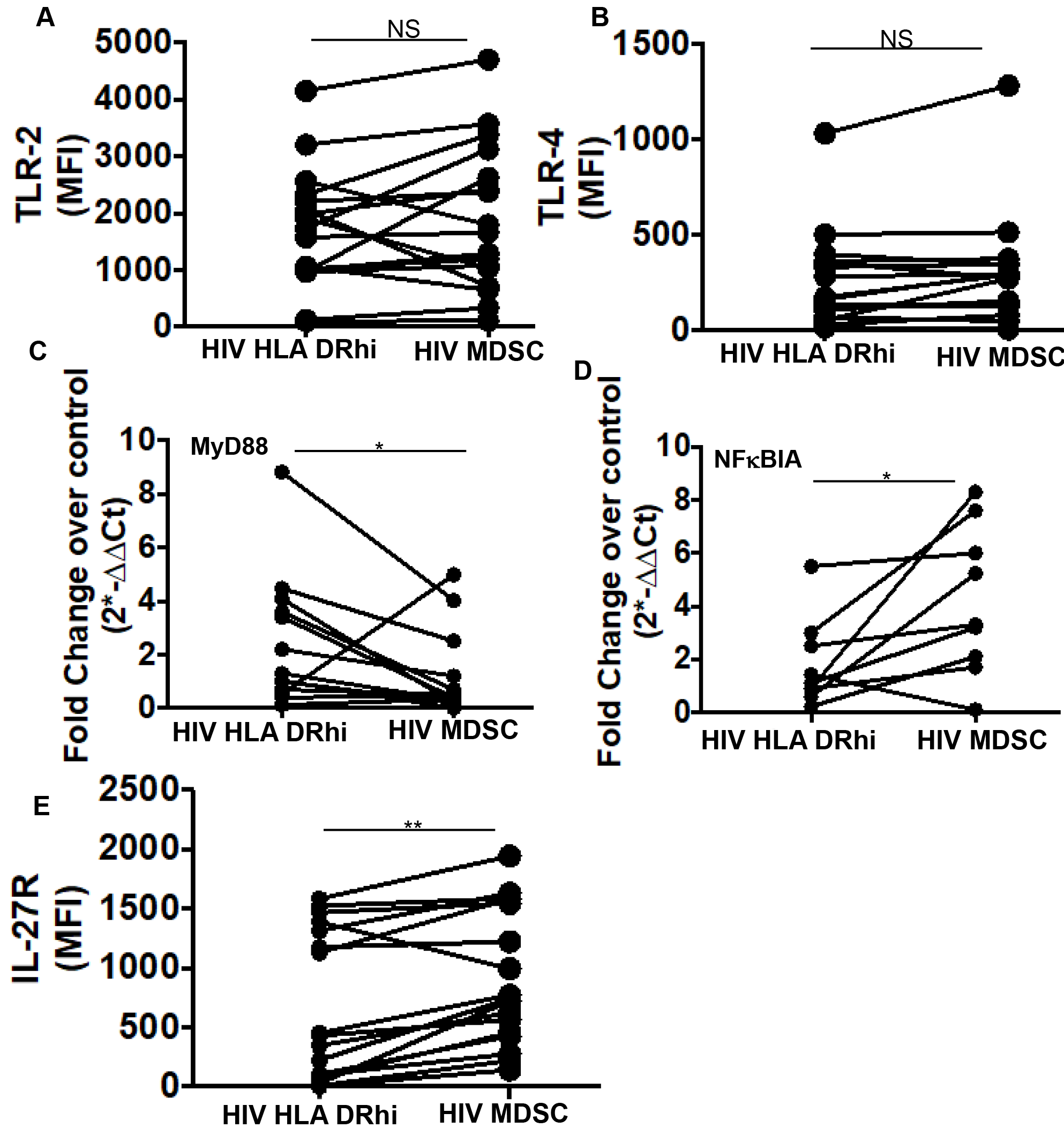
 Supplementary Figure 3

**(A-D) HIV MDSC as compared to HLA DR^hi^ cells exhibit truncated TLR signaling in response to *M tuberculosis*:** CD11b^+^CD33^+^CD14^+^HLA DR^+/hi^ (HIV HLA DR^hi^) and CD11b^+^CD33^+^CD14^+^HLA DR^-/lo^ (HIV MDSC) were stained with anti-TLR2 and anti-TLR4 antibodies. The expression of TLR-2 (A) and TLR-4 (B) was determined by flow cytometry and Mean Fluorescence Intensity (MFI) calculated, and (C-D) cultured without or with *M tuberculosis* WCL for 24 hours, expression of housekeeping gene 18S RNA and MyD88 (C) and NFκBIA (D) and was determined using TaqMan Gene expression assay, and ∆∆C_T_= ∆C_T,WCL_-∆C_T,control_. The fold change was calculated as 2 ^- (∆∆C^_T_). (E) **HIV MDSC as compared to HLA DR^hi^ cells express higher level of IL-27R:** CD11b^+^CD33^+^CD14^+^HLA DR^+/hi^ (HIV HLA DR^hi^) and CD11b^+^CD33^+^CD14^+^HLA DR^-/lo^ (HIV MDSC) were stained with anti-IL-27R antibody. The expression of IL-27R was determined by flow cytometry and Mean Fluorescence Intensity (MFI) calculated. For all figures, each dot represents an individual donor *p<0.05, **p<0.005, NS: Non-Significant.


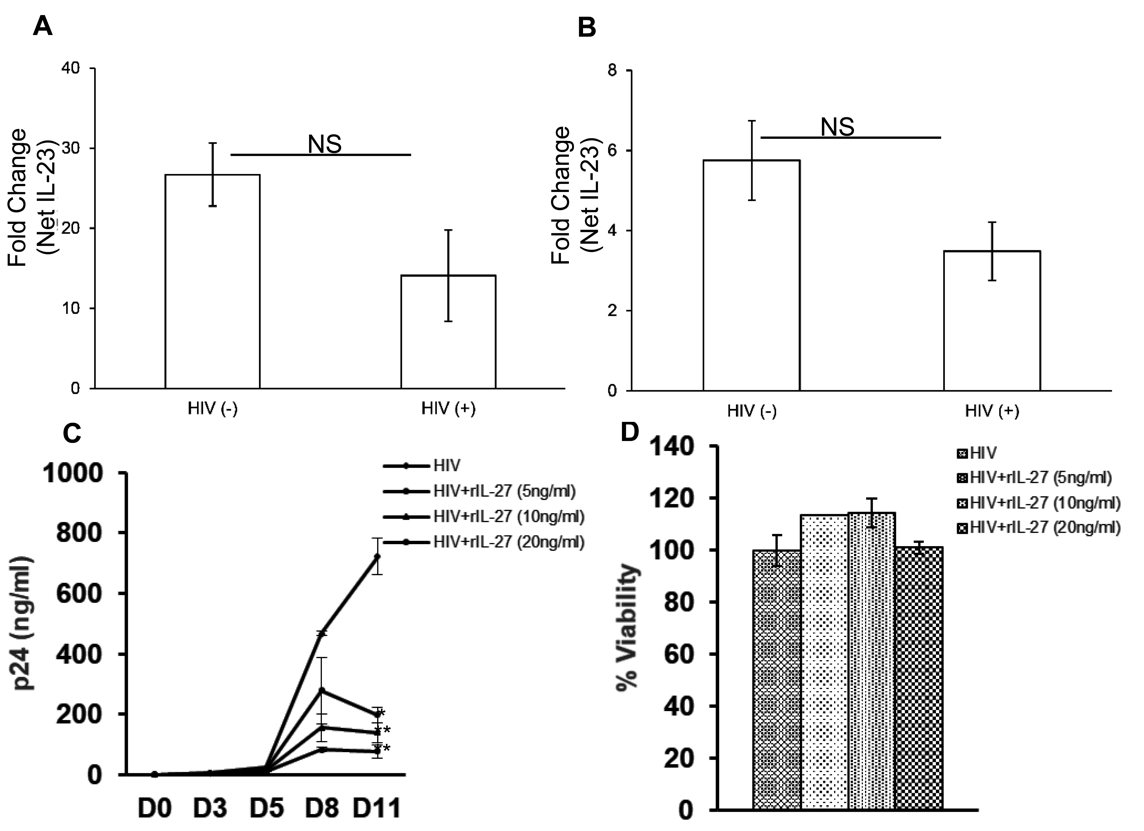
Supplementary Figure 4

**(A-B) HIV MDSC express low levels of IL-23 in response to *M tuberculosis*:** CD11b^+^CD33^+^CD14^+^HLA DR^-/lo^ (MDSC) from HIV-infected individuals on combined anti-retroviral therapy and virologic suppression were and HIV (-) healthy controls were (A) infected with *M tuberculosis* Erdman or (B) cultured with *M tuberculosis* whole cell lysate for 24-48 hours. The quantities of IL-23 was measured by Luminex multiplex cytokine assay. **(C-D)** **IL-27 inhibits HIV replication:** PHA stimulated PBMCs of HIV (-) donors were infected with infectious HIV_BaL_ (MOI 0.01) in presence of IL-2 with or without rIL-27 (5, 10 and 20 ng/ml). Culture supernatants were collected at indicated time post-infection. (C) Amount of p24 as an indicator of HIV replication was quantitated by ELISA. (D) Cytotoxicity of PBMCs at day 11 post-infection was determined by LDH release assay. Cells from each donor were infected in triplicates. For A and B, each dot represents an individual donor; for C and D, mean values +/- SEM are shown from 3 donors. *p<0.05, NS: Not Significant
